# Self-Centering Steel–Timber Hybrid Shear Wall: Experimental Test and Parametric Analysis

**DOI:** 10.3390/ma13112518

**Published:** 2020-06-01

**Authors:** Ye Cui, Fei Chen, Zheng Li, Xiaojuan Qian

**Affiliations:** 1 Department of Disaster Mitigation for Structures, Tongji University, Shanghai 200092, China; 2010_yecui@tongji.edu.cn; 2 Department of Structural Engineering, Tongji University, Shanghai 200092, China; zhengli@tongji.edu.cn; 3 JiangXi GuoJin Green Construction Technology Co., Ltd., Nanchang 330100, China; judy@jinheng-sh.com

**Keywords:** steel–timber hybrid structure, earthquake resilience, self-centering, slip friction damper, parametric analysis

## Abstract

Due to timber material’s environmental benefits and satisfactory structural properties, the studies on providing solutions to the application of timber to mid-rise or even high-rise buildings have been recently increasing. Among them, the steel–timber hybrid shear wall (STHSW) is one of the promising lateral resisting systems. However, the application of the system is limited because of its unsatisfactory earthquake resilience. In this paper, a new system, self-centering (SC)-STHSW, is proposed by introducing post-tensioned (PT) technology into the STHSW system. The cyclic loading test of one full-scale SC-STHSW specimen was conducted. The new system was proved to have both satisfactory self-centering capacity and the sufficient energy dissipation. Within the OpenSees platform, a numerical model was developed and validated by the experiment result. The model was further used in the parametric analysis. The system’s self-centering capacity, energy dissipation performance and the ultimate strength were evaluated under multiple parameters. The parameters included the initial PT stress ratio, the relative value of the damper’s activation force, the wood shear wall’s resistance, the beam section height and the wood shear wall’s strength. The lateral wall-to-frame stiffness ratio was also considered. Each parameter’s effects on three different performances of the system were analyzed. Based on the analysis results, a design parameter, a self-centering ratio, was proposed. The ratio was suggested to be larger than 0.5 to ensure a favorable self-centering performance in the system. This study provides support to the application of the innovative steel–timber hybrid structural system in practical engineering.

## 1. Introduction

Wood is one kind of environment-friendly material [1,2]. It has a high strength-to-weight ratio, high thermal insulation ability and requires low energy consumption during the production. With the advantages of wood material, timber structure has been earning increasing attention in recent decades. A lot of research has been focused on making timber structure more applicable to mid-rise or even high-rise buildings [3,4,5,6,7]. The hybridization between timber and steel material is one of the research hotspots [8].

For the shear wall system, Tesfamariam et al. [9] proposed a hybrid steel–timber system. It is composed of a steel frame and a cross-laminated timber (CLT) shear wall. The hybrid system was taken as the lateral resistance of a nine-story building. Through nonlinear time history analysis, it was found that the peak inter-story drift of the building was effectively controlled to lower than 2%. To improve the in-plane resistance of the timber platform-frame shear wall, Trutalli et al. [10] proposed a new hybrid system. The system is composed of steel columns, timber beams and oriented strand board (OSB) panels. Through cyclic loading tests on a full-scale specimen, the system was proved to have satisfactory in-plane strength and ductility. An innovative timber shear-wall system was proposed by Scotta et al. [11]. A technoprene plaster-infilled slab was incorporated into the light-frame wood shear wall. According to the experimental result, it was found that a high ductility and energy dissipation capacity were available in the system. He et al. [12] proposed an innovative steel–timber hybrid shear wall (STHSW) system. The system is composed of the light frame wood shear wall and the steel frame. The two subassemblies are connected by the bolted connection. According to the cyclic loading test result, the light frame wood shear wall significantly increased the stiffness of the system and postponed the yielding of the steel frame. To predict the behavior of the STHSW system, Li et al. [13] developed a detailed ABAQUS model. The accuracy of the numerical model was validated against the test result. To further change the energy dissipation mechanism, Li et al. [14] changed the bolted connection of the STHSW system to one innovative slip friction damper. The work of the friction damper was designed to be three phases, including the before activation phase, the activation phase and the lock-up phase [15]. Three STHSW specimens with the slip friction dampers were taken to conduct the hysteretic loading test by Li et al. [14]. According to the test result, the energy dissipation of the STHSW system was successfully transferred from the nails’ yielding to the sliding friction energy in the dampers.

However, a large residual displacement was observed after the test. The lock-up of the dampers led to the accumulation of the damage in the wood shear wall. Extra restoring action is needed in the STHSW system. Post-tensioned (PT) steel frame has been studied since 2001 [16,17,18,19,20,21,22]. The PT connection is successfully used as the beam-to-column connection to provide a restoring ability to the steel frame. Recently, the study on the PT steel frame has been focused on the introduction of extra resistance into the frame system. The PT steel frame can therefore be used in high-rise buildings with large spans [23,24,25,26].

**Figure 1:**
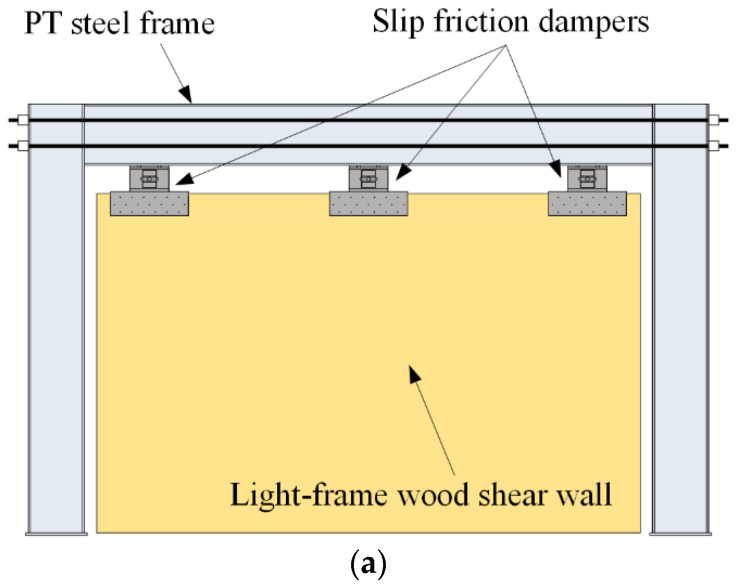

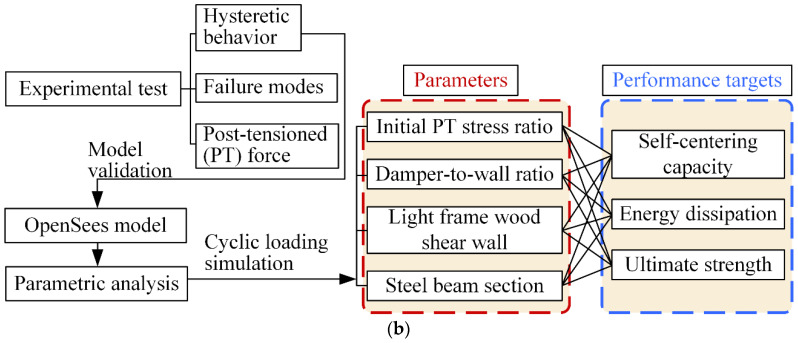
(**a**) Self-centering steel–timber hybrid shear wall (SC-STHSW); and (**b**) the flow chart of the research approach.

Considering the high resistance of the STHSW system and the need for extra restoring action, a new system, self-centering steel–timber hybrid shear wall (SC-STHSW), is proposed in this paper. As shown in Figure 1a, the system is composed of the PT steel frame and the light frame wood shear wall. The slip friction dampers are used as both the frame-to-wall connectors and the energy dissipaters.

The flow chart of the study’s research approach is shown in Figure 1b. In the first part of the paper, the new system’s hysteresis response and failure modes were analyzed based on the experimental result. Then, in the second part, a numerical model within the OpenSees platform was developed for the new system. After the accuracy validation of the developed model, the model was used to do the parametric analysis. Four parameters and three performance targets were used in the analysis. The influences of the different parameters on the performance of the SC-STHSW system were explored.

## 2. Experimental Test

One full-scale specimen of the SC-STHSW system was taken to the cyclic loading test. The details on the specimen configuration and the test arrangement will be introduced in the first subsection. Then, the failure modes observed in the test will be presented, followed by the evaluation of the hysteretic response of the SC-STHSW system.

### 2.1. Experiment Description

#### 2.1.1. Specimen

**Figure 2:**
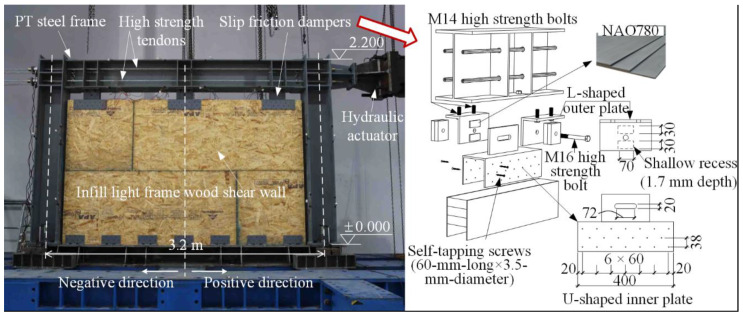
Configuration of the specimen.

As shown in Figure 2, the specimen tested was 3.2 m in width and 2.2 m in height. There were two subassemblies in the specimen, namely the PT steel frame and the light frame wood shear wall. They were connected by three slip friction dampers.

In the PT steel frame, H340 × 250 × 9 × 14 (i.e., 340 mm in height, 250 mm in width, 9 mm in web thickness, and 14 mm in flange thickness) and H300 × 300 × 10 × 15 were used for the beam and column section, respectively. The steel grade was Q235B, conforming to Chinese Standard GB 50017 [27]. In each side of the web of the steel beam, two high-strength tendons passed through the prefabricated holes. They were anchored at the columns after the post-tensioning process. Each of the four tendons was with a diameter of 15.2 mm and a yield strength of 1674 MPa. The initial stress of each strand was designed as 30% of the yield strength. By multiplying the initial stress by the total section area of the four tendons (560 mm^2^), the initial PT force obtained was 280 kN.

The light frame wood shear wall was made of a wooden frame and four sheathing panels. In the wooden frame, there were vertical studs and horizontal plates as the frame members. They were made of dimension lumbers with a cross-section area of 38 × 89 mm. The dimension lumbers used in the test were produced from spruce–pine–fir (SPF) with a kiln-dried No.3 grade (according to National Lumber Grading Authority NLGA 2014 [28]). The dimension lumbers’ average moisture content was 20.3%. The high moisture content might have been related to the laboratory’s environment. As to the sheathing panels, four oriented strand board (OSB) panels were used. The panel thickness was 9.5 mm. Additionally, 82.5 mm long × 3.3 mm diameter nails were used to connect the panel with the frame members. As to the sheathing-to-framing connections, the spacing was 75 mm along the sheathing edge and 150 mm along with the intermediate supports of the sheathings. The wood shear wall was bolted to the foundation beam through fourteen steel angles. The vertical leg of each steel angle was connected to the wall by the self-tapping screws (60 mm long × 3.5 mm diameter).

Three slip friction dampers were used in the test. Each of them was made of one inner plate (U-shape), two outer plates (L-shape), and four friction pads. Q235 grade steel was used for the inner and outer plates. Non-asbestos organic 780 (NAO780) material was used for the friction pads. A 12.9 grade M16 high-strength bolt was used to clamp the plates and friction pads together. The tightening torque was set as 140 N·m. According to [15], the activation force of each damper was 15 kN. As shown in Figure 2, there was a slot hole in the inner plate. It was designed to realize the relative motion between the PT steel frame and the wood shear wall. Along one direction, the maximum value of the relative motion was half of the slot length. The slot length was set as 72 mm in the test. It was determined by the 1.6% inter-story drift. In each of the dampers, M14 high-strength bolts were used to connect the outer plate to the steel beam. The inner plate was connected to the wood shear wall with self-tapping screws (60 mm long × 3.5 mm diameter).

#### 2.1.2. Loading Protocol and Data Measurement

**Figure 3:**
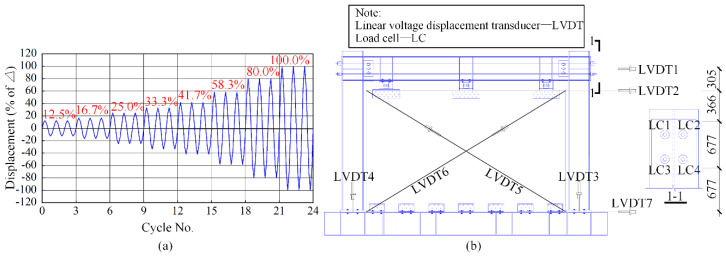
Test arrangement: (**a**) the loading protocol; (**b**) the data acquisition (all dimensions are in mm).

The American Concrete Institute ACI Innovative Task Group 5 [29] recommended a loading protocol with the displacement control pattern for the post-tensioned structures. The loading protocol was adopted in the test, as shown in Figure 3a. The controlled displacement *Δ* was set as 66 mm in the test, which corresponded to an inter-story drift of 3%. Linear voltage displacement transducers (LVDTs) were used to obtain the deformation of the SC-STHSW specimen. As shown in Figure 3b, seven LVDTs were used. The lateral deformation of the specimen and the wood shear wall were recorded by LVDT 1 and LVDT 2. LVDT 3 and LVDT 4 were used to record the uplift deformation at the two column bases. LVDT 5 and LVDT 6 were diagonally placed to record the shear deformation of the wood shear wall. LVDT 7 was placed at the top face of the foundation beam to check the possible displacement. Four load cells (LCs) were installed at each of the tendons to record the PT force variation in each tendon.

### 2.2. Experiment Result

#### 2.2.1. Hysteretic Response and Failure Modes

**Figure 4:**
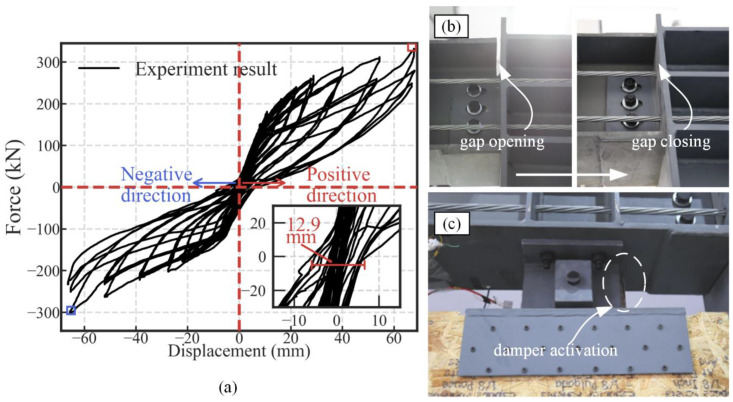
(**a**) Hysteretic response of the SC-STHSW specimen; (**b**) the gap opening and closing in the PT connection; (**c**) the activation of the slip friction damper.

The hysteretic response of the SC-STHSW specimen is shown in Figure 4a. The force value was taken as the actuator force, while the displacement was taken as the data from LVDT 1. The general shape of the hysteresis curve was a flag shape. It was similar to the peculiar response from the self-centering steel frame as reported in [20,30,31], which means that the satisfactory restoring and energy dissipation capacity were obtained in the SC-STHSW system. Compared with the hysteresis curve of the STHSW system reported in [14], the largest residual deformation of the SC-STHSW specimen was only 12.9 mm. The satisfactory restoring capacity was due to the successful gap opening and closing (Figure 4b) in the beam-to-column connections. In the test, the three slip friction dampers were activated (Figure 4c) around the displacement of 20 mm. The sliding friction in these dampers provided sufficient energy to the specimen. In Figure 4a, it was observed that there was an increase in the stiffness and the lateral force at the displacement of 40 mm. The reason was that the lock-up of the friction dampers forced the shear wall back to work with the PT frame, which led to the increase. The ultimate load at the positive and negative loading directions, labeled as a red square and a blue square, respectively, was 331.25 kN and −302.77 kN. The hysteretic curve of the specimen was asymmetrical. It could be explained that the bolt in the slot of the damper could not return to the original position after each loading cycle. The SC-STHSW specimen had an efficient self-centering ability, while the energy dissipation was simultaneously sufficient.

**Figure 5:**
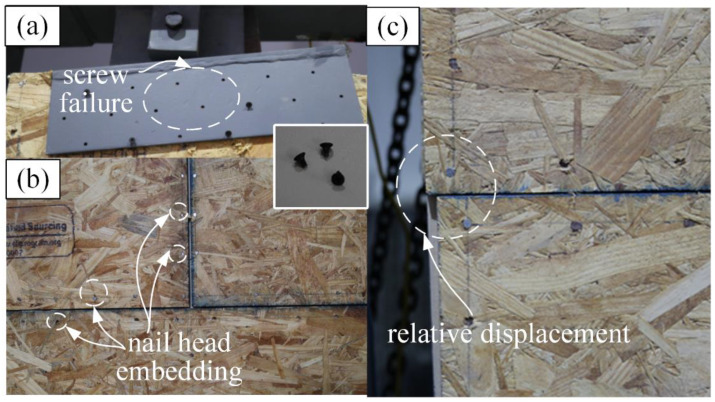
Failure modes: (**a**) the screw failure in the connection; (**b**) the nail head embedding; (**c**) the relative displacement between the panels.

After the test, it was observed that the serious damage was at the connection between the dampers and the wood shear wall. As shown in Figure 5a, the heads of many self-tapping screws in the connection were stripped. It is understandable since the dampers were also the shear connectors in the specimen. A large amount of shear force was accumulated in the three dampers, so the deformation between the PT frame and the wood shear wall could be coordinated. In addition, there were only minor damages observed in the wood shear wall. In the corner of the sheathing panels, the nail head embedding (Figure 5b) and the relative displacement between panels (Figure 5c) were observed. As to the common failure modes, e.g., nail pull-through, nail withdrawal and the pull-out of the vertical studs, as reported in [12,32,33,34,35], they were not observed in this test. The yielding in the PT frame was also not observed.

The better performance of the slip friction damper was available if the capacity design method [36] was used. For illustration, the application of the capacity design method is presented.

**Figure 6:**
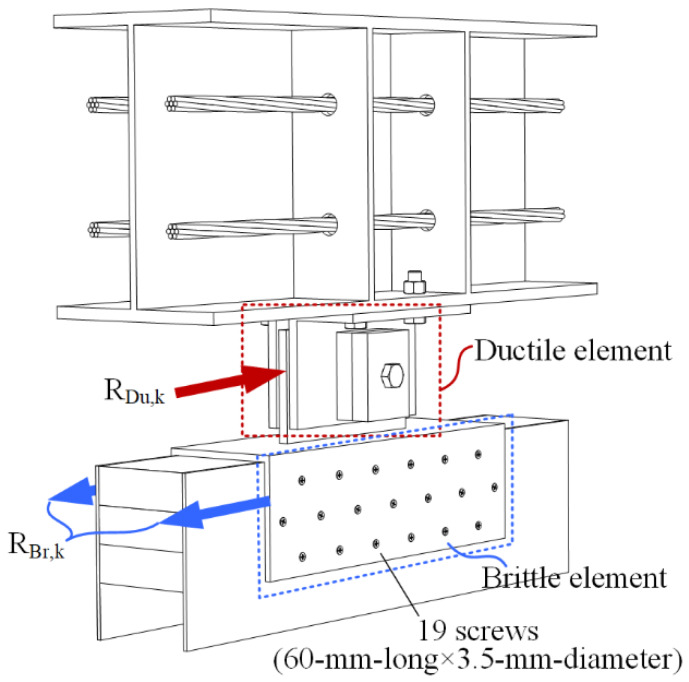
Schematics of one slip friction damper.

In each slip friction damper (Figure 6), the damper-to-wall connection was the brittle element. The plastic deformation in the damper could be fully utilized if an overstrength value was used, as given by Equation (1). The characteristic value is used in Equation (1) instead of the design value. The reason was that the partial material factor (*γ_M_*) was taken as 1.0, according to Eurocode 8 [37]. As shown in Figure 6, the thickness of the inner plate was 6 mm. It was larger than the diameter of the self-tapping screw (3.5 mm). Therefore, the steel plate was taken as the thick plate, according to Eurocode 5 [38]. The corresponding expression was used to calculate the characteristic strength of the damper-to-wall connection (*R*_Br,k_) as given by Equation (2). The required parameters are listed in Table 1. The slip friction damper’s overstrength value (*γ*_Rd_) was taken as 2.0, according to [39].

*R*_Br,k_ was calculated as 41.8 kN. Since the overstrength value was 2.0, the upper limit of the characteristic value of the friction force in the damper (*R*_Du,k_) was 20.9 kN. The value is 39% higher than the activation force (15 kN) of each damper used in the test. It means that before the activation of the dampers, the damper-to-wall connection was sufficiently rigid. However, the lock-up of the dampers increased the requirement on the resistance of the damper-to-wall connection. In the design of the slip friction damper, the characteristic value of the friction force (*R*_Du,k_) can be taken as a larger value. In turn, the screw quantity was increased to ensure the rigid connection in the damper-to-wall connection:

(1) $$R_{\mathrm{Br},k}\geq \gamma_{\mathrm{Rd}}\cdot R_{\mathrm{Du},k}$$

(2) $$R_{\mathrm{Br},k}=n\cdot \min\{\begin{array}{c} f_{h,k}t_{1}d\left[\sqrt{2+\frac{{4M}_{y,\mathrm{Rk}}}{f_{h,k}{dt}_{1}^{2}}}-1\right]+\frac{F_{\mathrm{ax},\mathrm{Rk}}}{4}\\ 2.3\sqrt{M_{y,\mathrm{Rk}}f_{h,k}d}+\frac{F_{\mathrm{ax},\mathrm{Rk}}}{4}\\ f_{h,k}t_{1}d \end{array}$$

where *R*_Br,k_ is the characteristic strength of the damper-to-wall connection; *γ*_Rd_ is the overstrength value of the slip friction damper; *R*_Du,k_ is the characteristic value of the friction force in the damper; *f*_h,k_ is the characteristic embedment strength in the timber member; *t*_1_ is the thickness of the steel plate; *d* is the screw diameter; *M*_y,Rk_ is the characteristic screw yield moment; and *F*_ax,Rk_ is the characteristic withdrawal capacity of the screw.

#### 2.2.2. Variation of PT Force

**Figure 7:**
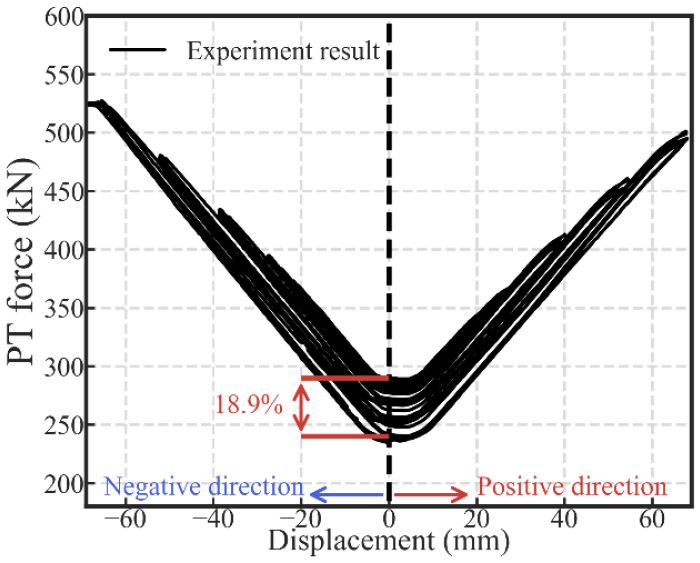
The variation of the PT force in the specimen.

The variation of the PT force in the four tendons is illustrated in Figure 7. The PT force is the sum of the data from the four load cells. The displacement was taken as the data from LVDT 1. As shown in Figure 7, the actual PT force initially applied to the specimen was 289.85 kN. It is 3.5% higher than the design value (280 kN), which is acceptable. When the lateral displacement was up to 60 mm in the negative direction, the PT force was around 500 kN. Therefore, the stress in each tendon was 892.86 MPa if the stress in each tendon was assumed to be the same. It is 0.53 times the yield strength (1674 MPa) of the high-strength tendon. The substantial growth of the PT force ensured the successful gap closing in the beam-to-column connection. The PT force loss is 18.9%, as shown in Figure 7, which is close to the loss of the PT force in [25,41,42,43].

## 3. Parametric Analysis

In this section, the influences of different parameters on the system’s performance were explored. Within the OpenSees platform, a numerical model was developed and validated by the experimental result. Subsequently, a parametric analysis was conducted after a selection of proper parameters.

### 3.1. Numerical Model

**Figure 8:**
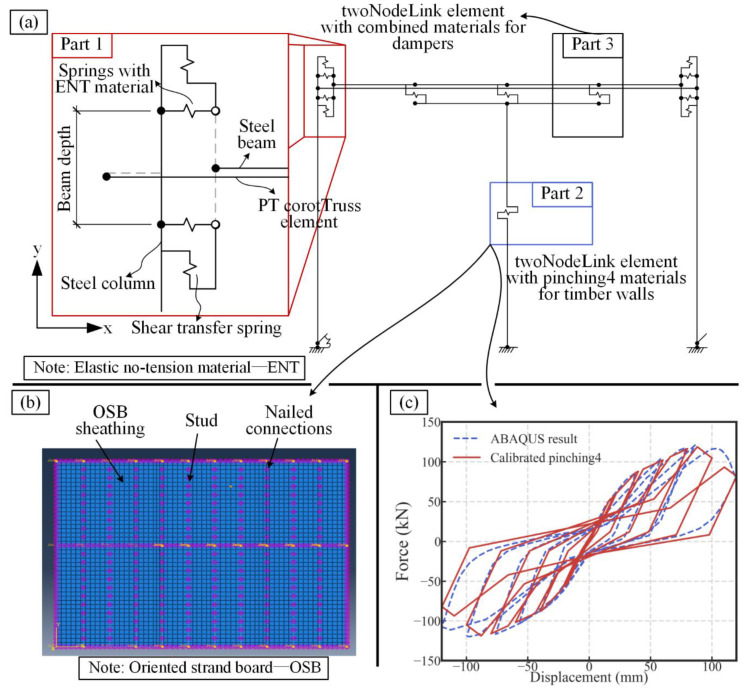
(**a**) Numerical model; (**b**) the detailed ABAQUS model for the light frame wood shear wall; (**c**) the calibration of the pinching4 material.

The developed numerical model is shown in Figure 8a. It has the same geometric dimension and material properties as the experiment specimen. As shown in Figure 8a, there are three main parts of the developed model. The first part is the PT connection. In the connection, the displacement-based beam-column element was used for the steel beam and columns. Two pairs of springs were used to simulate the gap opening in the PT connection at the top and toe of the beam section. The elastic no-tension material (ENT) was assigned to the two springs along the x direction. Two springs with elastic material were placed vertically in the connection to transfer the shear force. The corottruss2 element was used to represent the four high-strength tendons. The element material was steel02 that allowed the set of post-tensioning force. The simulation method used for the PT connection is commonly used in the study of PT steel frame [30,31,44,45]. The second part of the model is the light frame wood shear wall. A detailed numerical model was developed in ABAQUS (Figure 8b) based on the previous work by the authors [13,46]. After the cyclic loading, the hysteretic response was obtained from the ABAQUS model. It was used as the calibration source for the pinching4 material in OpenSees. During the calibration, the skeleton curve was determined based on the suggestion by [47]. The hysteretic rules were determined by the least-squares regression method. As shown in Figure 8c, the calibrated pinching4 material can satisfactorily reflect the hysteretic response obtained from the detailed ABAQUS model. The third part is the slip friction damper. The parallel springs were used to simulate the three working phases in the damper. The detailed numerical method is introduced in [15,48].

### 3.2. Model Validation

**Figure 9:**
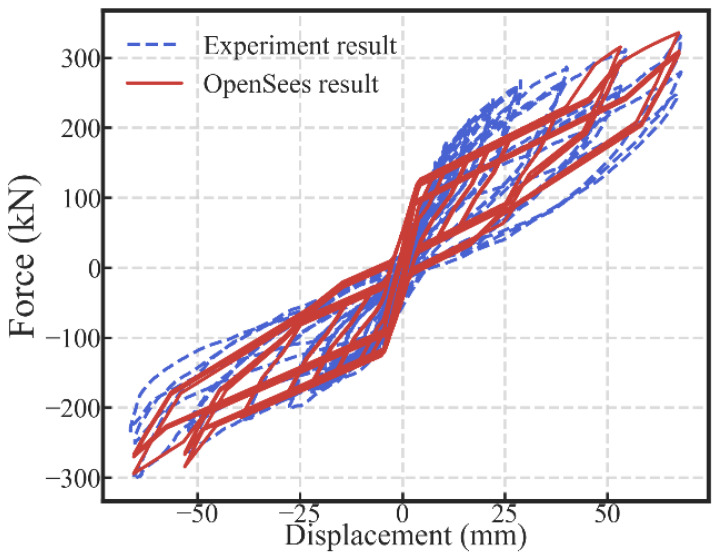
Model validation: the comparison of the hysteretic curves.

The comparison between the experimental result and the hysteretic response predicted by the model is shown in Figure 9. There was a strength deviation between the displacement of 20 and 40 mm along the positive direction. The reason was that the surfaces of the steel plates and friction pad in the damper were rough and uneven. Accordingly, there was a temporary lock-up of the dampers even after the damper activation. Some contribution to the strength was provided by the wall at the temporary lock-up of the dampers.

In general, the characteristic behavior, including the flag-shape hysteresis response and the second increase in the strength and stiffness of the SC-STHSW system were obtained by the developed model. The comparison verifies the accuracy of the developed model.

### 3.3. Parameters Selection

**Figure 10:**
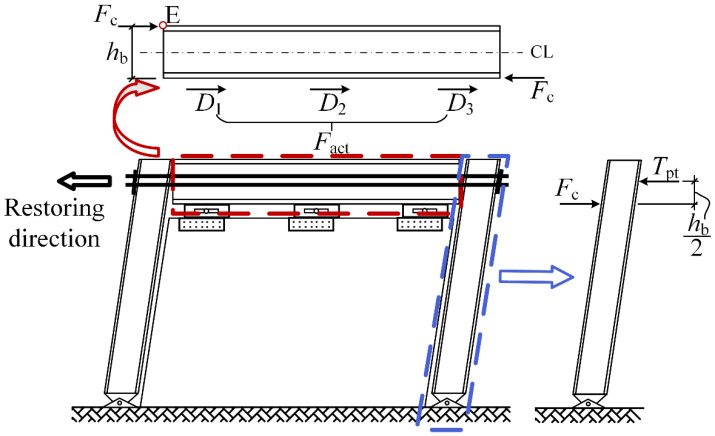
SC-STHSW system during the restoring process.

In Figure 10, the system during the restoring process was analyzed to select the parameters used for further analysis. In the free-body diagram of the steel beam, the two ends of the beam are under the compression force *F*_c_ from the columns. If assuming the column base was a hinged joint, the compression force *F*_c_ was close to the PT force (*T*_pt_) in the high-strength tendons. The reason is that the difference between the moment arms of the two forces were only half of the beam section height if we took a moment about the column base. At the bottom side of the steel beam, there were three friction forces (*D*_1_, *D*_2_, and *D*_3_) applied by the dampers. The sum of the three friction forces was the activation force (*F*_act_) of the dampers. By taking moments about point E, there were two moments (*M*_pt_ and *M*_f_) with inverse directions, as shown in Equations (3) and (4), respectively. The moment that helps the beam back to the origin position (*M*_pt_) was named the restoring moment. The other one (*M*_f_) was named as the anti-restoring moment. It was an obstacle to the restoring process of the steel beam. If the restoring moment (*M*_pt_) is always larger than the anti-restoring moment (*M*_f_), the self-centering performance of the system can be ensured to be satisfactory. It means that the PT force (*T*_pt_) should be larger than the activation force (*F*_act_) during the restoring process. In Equations (5) and (6), the PT force (*T*_pt_) and the activation force (*F*_act_) are further divided into the products of several basic parameters with the help of two dimensionless quantities, *κ* and *μ*. The first quantity *κ* is named as the initial PT stress ratio; and the second quantity *μ* is named as the damper-to-wall ratio. The two quantities are both in the range of 0–1:

(3) $$M_{\mathrm{pt}}{=T}_{\mathrm{pt}}\cdot h_{b}$$

(4) $$M_{f}{=F}_{\mathrm{act}}\cdot h_{b}$$

(5) $$\min\left\{T_{\mathrm{pt}}\right\}=\kappa \cdot f_{y}\cdot A_{\mathrm{pt}}$$

(6) $$F_{\mathrm{act}}=\mu \cdot F_{\mathrm{Wall}}$$

where *M*_pt_ is the restoring moment; *M*_f_ is the anti-restoring moment; *T*_pt_ is the PT force in the high-strength tendons; *F*_act_ is the activation force of the three dampers; *h*_b_ is the section height of the steel beam; *f*_y_ is the yield strength of the high-strength tendons; *A*_pt_ is the total cross section area of the high-strength tendons; *F*_Wall_ is the ultimate strength of the light frame wood shear wall.

Based on the simple analysis, the parameters related to the self-centering capacity of the SC-STHSW system are:

1. The initial PT stress ratio *κ*;
2. The damper-to-wall ratio *μ*;
3. The ultimate strength of the light frame wood shear wall *F*_Wall_;
4. The total cross-section area of the high-strength tendons *A*_pt_.

**Figure 11:**
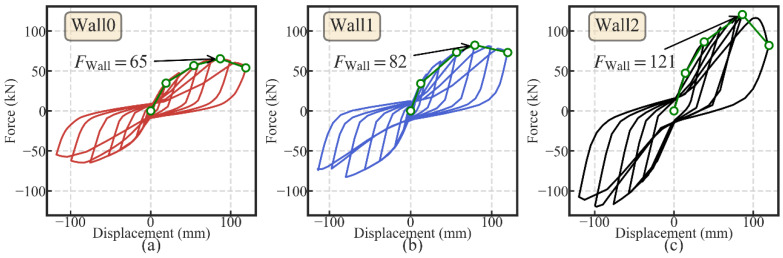
Hysteretic curves of the three light frame wood shear walls (**a**) wall 0; (**b**) wall 1; and (**c**) wall 2.

Among the four parameters, three were selected as the analysis parameters, including the initial PT stress ratio *κ*, the damper-to-wall ratio *μ* and the ultimate strength of the light frame wood shear wall *F*_Wall_. The section area *A*_pt_ was not taken as the parameter since it shares the same function with the initial PT stress ratio *κ.* Instead of varying the value of *F*_Wall_ directly, three light frame wood shear walls were taken to represent the variation of *F*_Wall_. As shown in Table 2, the three light frame wood shear walls have different nail spacing and sheathing panel configurations. The ultimate strength of each wood shear wall was obtained through the detailed ABAQUS model prediction. According to the prediction result, the hysteretic curve of each wood shear wall is shown in Figure 11.

**Figure 12:**
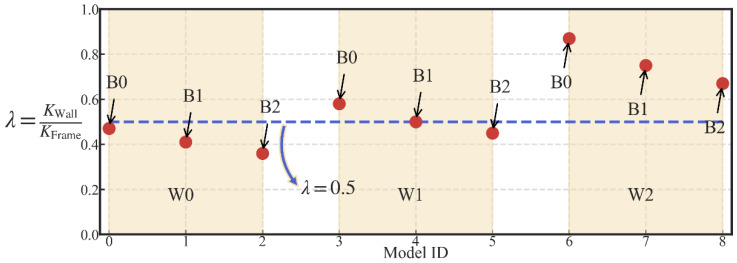
Lateral wall-to-frame stiffness ratio of the nine models.

In addition, the lateral wall-to-frame stiffness ratio *λ* has a significant influence on the performance of the STHSW system, according to [46]. Thus, three different beam sections were selected to create the different lateral wall-to-frame stiffness ratio *λ.* The beam sections selected are presented in Table 3. Three beam sections and three light frame wood shear walls made up nine models. The lateral wall-to-frame stiffness ratio *λ* of the nine models were shown in Figure 12. It can be seen that the ratio *λ* was controlled to vary around 0.5, which is the lateral wall-to-frame stiffness ratio suggested by [46].

In general, four parameters were selected for the parametric analysis, including the initial PT stress ratio *κ*, the damper-to-wall ratio *μ*, the ultimate strength of the light frame wood shear wall *F*_Wall_, and the steel beam section. In Table 4, the four parameters were presented. There were three beam sections and three light frame wood shear walls to construct nine SC-STHSW models. Each of the models had 13 levels of initial PT stress ratio and seven levels of the damper-to-wall ratio. Thus, a total of 819 models were considered for the parametric analysis. In these models, the column base was set as a hinged joint to meet the previously made assumption. Apart from that, the model’s other properties were the same as that of the model verified by the experiment.

**Figure 13:**
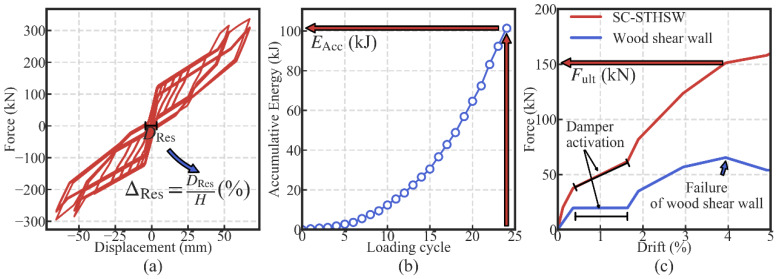
The definition of the performance targets: (**a**) the residual drift; (**b**) the accumulative energy dissipation; and (**c**) the ultimate strength.

In the SC-STHSW system, the self-centering capacity, energy dissipation and the ultimate strength are important. They were the performance targets in the parametric analysis. The first two targets, the self-centering capacity and the energy dissipation, were obtained by a cyclic loading simulation. The cyclic loading simulation was conducted for all 819 models. The loading protocol used was the same as that of the experiment. The index selected to evaluate the self-centering capacity was the residual drift $\Delta_{\mathrm{Res}}$. It was obtained by dividing the largest residual displacement (*D*_Res_) by the height of the specimen (2200 mm), as shown in Figure 13a. The low value of $\Delta_{\mathrm{Res}}$ indicates a satisfactory self-centering capacity of the SC-STHSW system. In Figure 13b, the accumulative energy dissipation under the last loading cycle ($E_{\mathrm{Acc}}$) was used to evaluate the system’s energy dissipation performance. As to the ultimate strength, a pushover analysis was conducted for all 819 models. The force, corresponding to the wood shear wall’s failure, was taken as the ultimate strength ($F_{\mathrm{ult}}$), as shown in Figure 13c. There is still an increase in force after the failure of the wood shear wall. However, it is not considered as the ultimate strength because the wood shear wall is taken as the main lateral resistance in the SC-STHSW system.

### 3.4. Analysis Results

**Figure 14:**
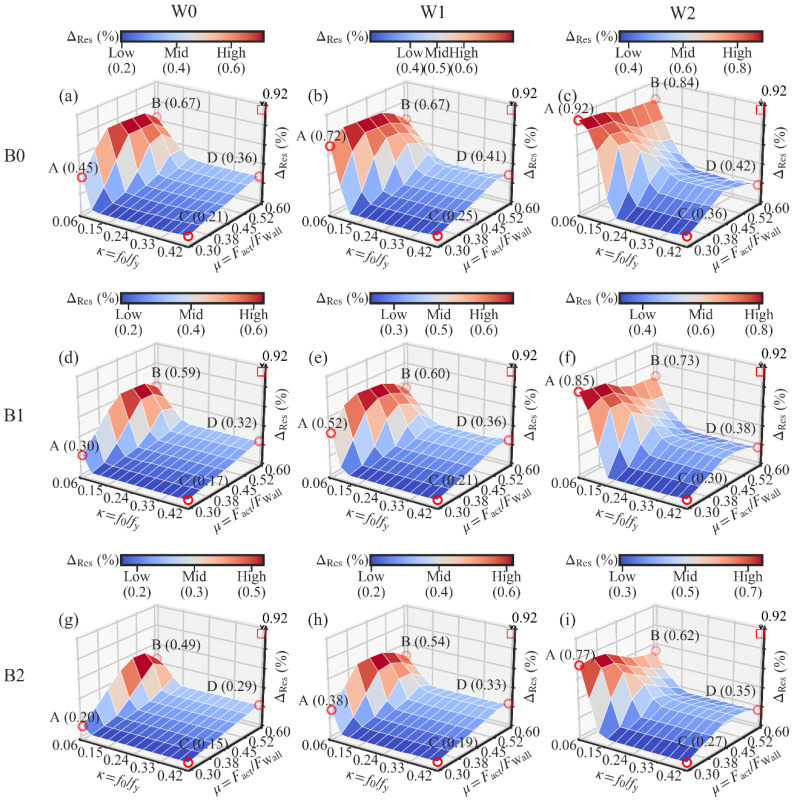
Three-dimensional surfaces of the residual drift: (**a**) W0B0; (**b**) W1B0; (**c**) W2B0; (**d**) W0B1; (**e**) W1B1; (**f**) W2B1; (**g**) W0B2; (**h**) W1B2; (**i**) W2B2.

**Figure 15:**
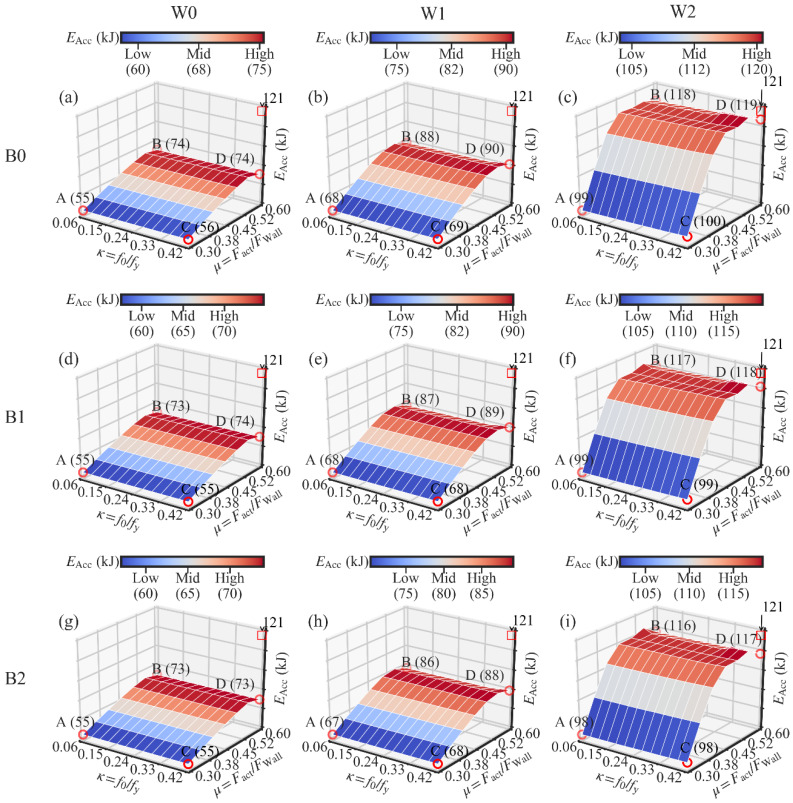
Three-dimensional surfaces of the energy dissipation: (**a**) W0B0; (**b**) W1B0; (**c**) W2B0; (**d**) W0B1; (**e**) W1B1; (**f**) W2B1; (**g**) W0B2; (**h**) W1B2; (**i**) W2B2.

**Figure 16:**
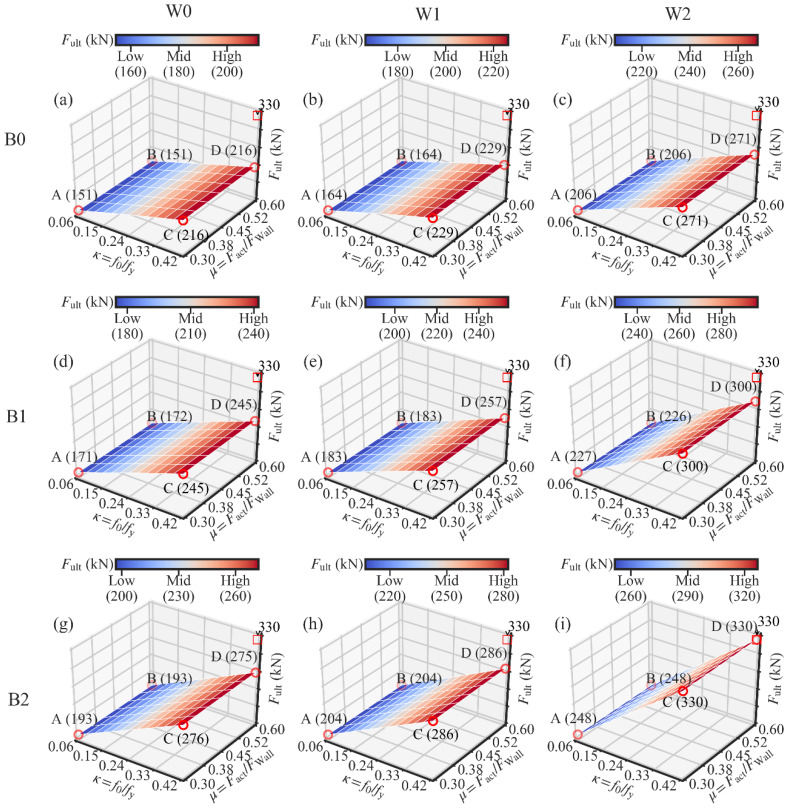
Three-dimensional surfaces of the ultimate strength: (**a**) W0B0; (**b**) W1B0; (**c**) W2B0; (**d**) W0B1; (**e**) W1B1; (**f**) W2B1; (**g**) W0B2; (**h**) W1B2; (**i**) W2B2.

The analysis results are presented in Figure 14, Figure 15 and Figure 16. Each figure corresponds to the analysis results of one performance target.

In Figure 14, the performance target is the self-centering performance. The simulation results are presented by nine 3D surfaces. Each of the surfaces corresponds to one combination of the beam section and the light frame wood shear wall. The three axes on each surface are kept the same for comparison. The vertical axis’ upper value is the largest residual drift $\Delta_{\mathrm{Res}}$ of all 819 models, which was 0.92%, as shown in each subfigure. Four points (A, B, C and D) were labeled in the four corner points of each subfigure. Each of them corresponds to an extreme set of the initial PT stress ratio *κ* and the damper-to-wall ratio *μ*. In Figure 15 and Figure 16, the energy dissipation and ultimate strength are the performance targets, respectively.

#### 3.4.1. Influences of the Initial PT Stress Ratio

**Figure 17:**
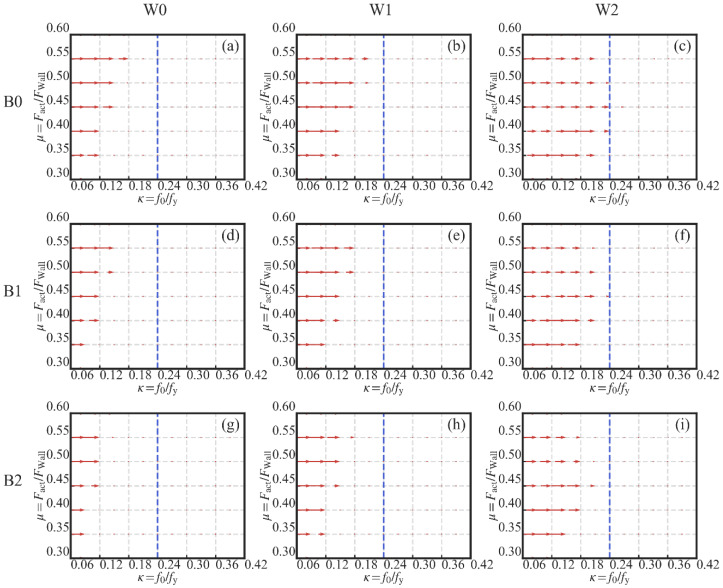
Gradient field (along the direction of the decrease in *κ*): (**a**) W0B0; (**b**) W1B0; (**c**) W2B0; (**d**) W0B1; (**e**) W1B1; (**f**) W2B1; (**g**) W0B2; (**h**) W1B2; (**i**) W2B2.

The influence of the initial PT stress ratio *κ* on the residual drift $\Delta_{\mathrm{Res}}$ is similar in the nine subfigures. Taking Figure 14a as an instance, it can be observed that the residual drift $\Delta_{\mathrm{Res}}$ drops down to a low value and then goes to a plateau section with the increase in *κ.* It is especially obvious along the side AC or side BD of the 3D surface. It means that the increase in *κ* has a positive influence on the decrease in $\Delta_{\mathrm{Res}}$. However, the influence is limited when *κ* is at a relatively high level. In Figure 17, the gradient vector field of each 3D surface was calculated using a second-order accurate central differences method. Each gradient vector is calculated along the direction of the decrease in *κ*. The length of each vector is in proportion to the gradient value. It is obvious that the influence of *κ* on $\Delta_{\mathrm{Res}}$ is very weak when *κ* is larger than 0.24. It is suggested that the lower limit value of *κ* is 0.24 to guarantee the maximum effectiveness of the PT high-strength tendons. As to the upper limit value of *κ*, the value larger than 0.4 is not suggested based on the experiment result. In the experiment, the PT stress was up to 0.53 times the yield strength under the largest lateral deformation. *κ* is not suggested to be larger than 0.4 to avoid the development of plasticity in the high-strength tendons.

As shown in Figure 15, there is no influence of the variation of the initial PT stress ratio *κ* on the energy dissipation ($E_{\mathrm{Acc}}$). This is understandable since the PT high-strength tendons are introduced to provide the self-centering capacity to the system, instead of energy dissipation capacity. In addition, the lack of influence of *κ* on the energy dissipation shows that the tendons remained elastic during the simulation.

In Figure 16, it can be observed that the increase in the ultimate strength ($F_{\mathrm{ult}}$) is almost linear to the increase in *κ*. Taking the side AC of Figure 16a as an instance, $F_{\mathrm{ult}}$ increases from 151 to 216 kN when *κ* increases from 0.06 to 0.42. Since the same wood shear wall was used in Figure 16a, the increase in $F_{\mathrm{ult}}$ can only be attributed to the strength’s increase in the PT steel frame. It means that the increase in *κ* leads to the strength’s increase in the PT steel frame. This result is also reported by [49].

#### 3.4.2. Influences of the Damper-to-Wall Ratio

In Figure 14a,d,g, the increase in the damper-to-wall ratio *μ* is almost linear to the increase in the residual drift $\Delta_{\mathrm{Res}}$ when the initial PT stress ratio *κ* is larger than 0.15. The increase in *μ* leads to the increase in the activation force of the dampers, which is an obstacle to the restoring of the SC-STHSW system. In Figure 14b,c,e,f,h,i, a knee point is observed along the side CD. After a certain value of *μ*, $\Delta_{\mathrm{Res}}$ is not changed or even decreased with the further increase in *μ*. It can be explained by the activation failure of the dampers. With a high value of *μ*, the requirement of the damper activation is difficult to satisfy. The inactivated dampers can only apply force that is lower than the activation force to the steel beam. This means that there is less of an obstacle to the restoring of the SC-STHSW when the dampers are not activated than in the case where the dampers are activated. The explanation can be consolidated by putting the focus on the side CD of Figure 14b,e,h or Figure 14c,f,i. In Figure 14b,e,h, a knee point is observed when *μ* is around 0.52. The reason is that the same light frame wood shear wall is used in the three subfigures. In Figure 14c,f,i, the knee point is observed when *μ* is around 0.45. The value is lower than 0.52 since the wood shear wall shared by these three subfigures has larger ultimate strength than that of Figure 14b,e,h. The use of a wood shear wall with large ultimate resistance narrows the range of *μ* where the successful damper activation is available. In addition, the largest residual drift is not at point B in all nine subfigures of Figure 14. It can also be attributed to the activation failure of the dampers.

**Figure 18:**
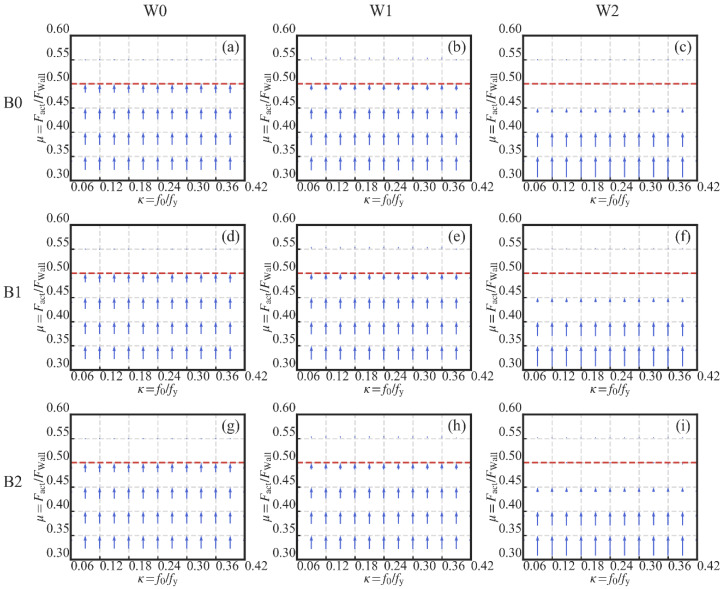
Gradient field (along the direction of the increase in *μ*): (**a**) W0B0; (**b**) W1B0; (**c**) W2B0; (**d**) W0B1; (**e**) W1B1; (**f**) W2B1; (**g**) W0B2; (**h**) W1B2; (**i**) W2B2.

The increase in *μ* has a positive effect on the increase in the energy dissipation, as shown in Figure 15. In Figure 15b,c,e,f,h,i, a plateau section is obvious. This means that the further increase in *μ* has a minor influence on the increase in $E_{\mathrm{Acc}}$. The reason is the activation failure of the dampers when *μ* is larger than a certain level, as mentioned previously. The level can be obtained in Figure 18. The gradient vector field of each 3D surface in Figure 15 was calculated, as shown in Figure 18. Each gradient vector was calculated along the direction of the increase in *μ*. The length of each vector is in proportion to the gradient value. When *μ* is larger than 0.5, the gradient vector is too small to be seen. The lower limit of *μ* is suggested as 0.5 if the energy dissipation’s increase is concerned.

The influence of *μ* on the ultimate strength ($F_{\mathrm{ult}}$) is not apparent, as shown in Figure 16, because the damper-to-wall ratio only determines the time when the wood shear wall begins to be protected. If the same wood shear wall is used, the system’s ultimate strength is not affected by the increase in *μ*.

#### 3.4.3. Influences of the Light Frame Wood Shear Wall

According to the previous analysis, the increase in the lateral resistance of the wood shear wall limits the selection range of the damper-to-wall ratio *μ* if the successful activation of the dampers is expected. On the other hand, the use of the wood shear wall with large resistance increases the $\Delta_{\mathrm{Res}}$ of the SC-STHSW system. In Figure 14a–c, the value of $\Delta_{\mathrm{Res}}$ at point A is increased from 0.45% (W0) to 0.92% (W1). It indicates that the increase in the lateral resistance of the wood shear wall shares the same function with the damper-to-wall ratio *μ* in decreasing the self-centering capacity of the SC-STHSW system. The negative effect of the wood shear wall with high resistance on the self-centering capacity is suggested to be considered in the SC-STHSW system’s design, especially in the case when the increase in lateral resistance of the system is needed.

In Figure 15, it is evident that the larger the wood shear wall’s lateral resistance, the larger is the energy dissipation obtained in the SC-STHSW system. The reason is that the increase in the wood shear wall’s lateral resistance increases the dampers’ activation force. The activation force is also the sliding friction force in the dampers. With the increase in friction force, the energy dissipated by the sliding friction is increased. Besides, the plateau section is longer in Figure 15c,f,i than that in Figure 15b,e,h. It reveals that the increase in the lateral resistance of the wood shear wall shares the same function with the damper-to-wall ratio *μ* in increasing the dampers’ activation force.

The increase in the wood shear wall’s lateral resistance has a positive effect on the system’s ultimate strength ($F_{\mathrm{ult}}$). Taking Figure 16a–c as an example, the ultimate resistance at point C increases from 216 kN (W0) to 271 kN (W2). The ultimate resistance is increased by 25% by using W2 instead of W0.

#### 3.4.4. Influences of the Steel Beam Section

The influence of the different beam sections on the residual drift $\Delta_{\mathrm{Res}}$ can be obtained by comparing the point A in Figure 14a,d,g. It is obvious that with the variation of the beam sections, the residual drift $\Delta_{\mathrm{Res}}$ is decreased from 0.45% (B0) to 0.2% (B2). Additionally, a 56% reduction is obtained. The variation of the beam sections is mainly related to the beam section height. It means that the increase in the beam section height contributes to the decrease in the residual drift $\Delta_{\mathrm{Res}}$. However, the influence of the beam section height on $\Delta_{\mathrm{Res}}$ is weaker at the high PT stress ratio than at the low PT stress ratio. This can be observed from the comparison between point C in Figure 14a,d,g. The residual drift $\Delta_{\mathrm{Res}}$ is decreased from 0.21% (B0) to 0.15% (B1). Only a 29% reduction is obtained. A similar observation is available in the comparison between Figure 14b,e,h or between Figure 14c,f,i. The improvement in the self-centering capacity of the SC-STHSW system is available if the beam section height is increased, especially when the initial PT stress ratio *κ* is at a low level.

In Figure 15, it is hard to see the influence of the steel beam section on the energy dissipation. However, the steel beam section’s effect on the ultimate strength cannot be ignored. In Figure 16 a,d,g, point A’s ultimate strength increases from 151 kN (B0) to 193 kN (B1). A growth of 28% is obtained by changing the beam section.

#### 3.4.5. Influences of the Lateral Wall-to-Frame Stiffness Ratio

**Figure 19:**
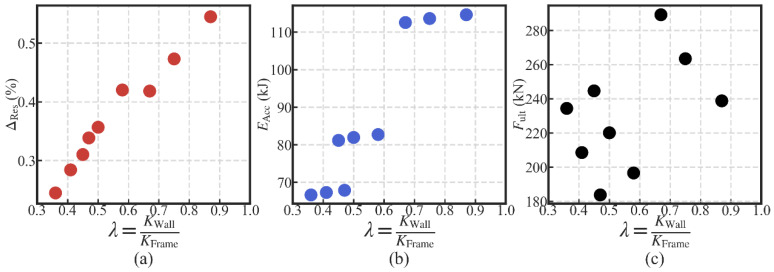
Influences of the lateral wall-to-frame stiffness ratio on: (**a**) self-centering performance; (**b**) energy dissipation performance; (**c**) ultimate strength.

The data in each subfigure of Figure 14, Figure 15 and Figure 16 were averaged to show the relationship between three performance targets with the lateral wall-to-frame stiffness ratio *λ*, as shown in Figure 19. With the increase in *λ*, the residual drift $\Delta_{\mathrm{Res}}$ increases. This means that the increase in *λ* negatively influences the self-centering capacity of the SC-STHSW. The energy dissipation ($E_{\mathrm{Acc}}$) is positively affected by the increase in *λ*. In this study, the wood shear wall’s variation is obtained by varying the nails’ spacing and sheathing configuration. This means that the wood shear wall with larger lateral resistance also has a higher stiffness, which in turn leads to a larger *λ*. It is the reason for the positive influence of *λ* on the energy dissipation. In Figure 19c, the ultimate strength ($F_{\mathrm{ult}}$) varies with the increase in *λ*. However, no obvious regularity is found.

#### 3.4.6. Self-Centering Ratio

**Figure 20:**
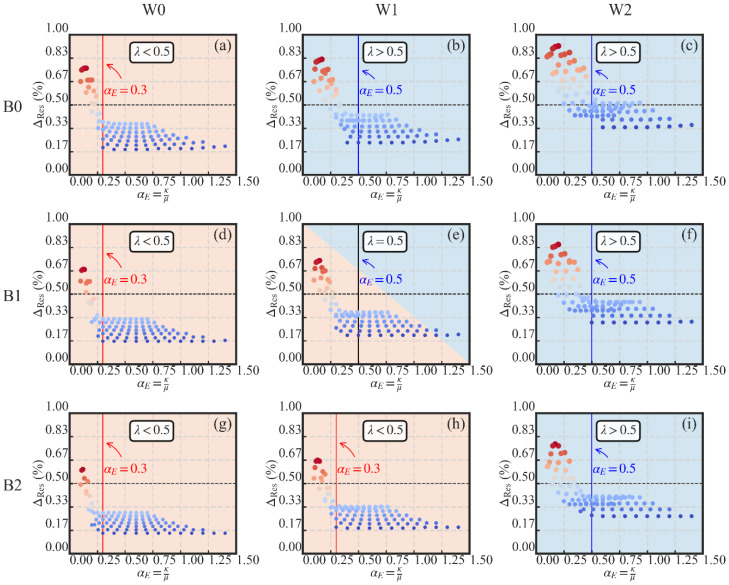
$\Delta_{\mathrm{Res}}$ versus $\alpha_{E}$ in: (**a**) W0B0; (**b**) W1B0; (**c**) W2B0; (**d**) W0B1; (**e**) W1B1; (**f**) W2B1; (**g**) W0B2; (**h**) W1B2; (**i**) W2B2.

Based on the previous analysis, the self-centering capacity of the SC-STHSW system is influenced by different parameters. For the convenience of the design, two of the parameters are combined together. A new parameter $\alpha_{E}$ is defined in Equation (7). It is named the self-centering ratio. In Figure 20, the relationship between the self-centering ratio $\alpha_{E}$ and the residual drift $\Delta_{\mathrm{Res}}$ is presented. The increase in the self-centering ratio $\alpha_{E}$ leads to the decrease in the residual drift $\Delta_{\mathrm{Res}}$, as shown in Figure 20. It is reasonable since the increase in the self-centering ratio $\alpha_{E}$ corresponds to the increase in the PT stress ratio *κ* or the decrease in the damper-to-wall ratio *μ*. Both situations have a positive effect on the decrease in $\Delta_{\mathrm{Res}}$ as introduced in Section 3.4.1 and Section 3.4.2. More importantly, there is a lower limit value for $\alpha_{E}$ to obtain a satisfactory performance in the self-centering of the SC-STHSW system. In the cases where the lateral wall-to-frame stiffness ratio *λ* is lower than 0.5 (Figure 20a,d,g,h), the lower limit value of $\alpha_{E}$ is 0.3. This value is increased to 0.5 if the lateral wall-to-frame stiffness ratio *λ* is larger than or equal to 0.5 (Figure 20b,c,e,f,i). When $\alpha_{E}$ is controlled to be larger than the lower limit value, the residual drift $\Delta_{\mathrm{Res}}$ of the SC-STHSW system is controlled into a relative stage value that is lower than 0.5%, which is suggested by [50] as the permissible residual deformation levels:

(7) $$\alpha_{E}=\frac{\kappa }{\mu }$$

## 4. Discussion

The new system, SC-STHSW, was proposed to overcome the large residual displacement of the STHSW system [12,14]. In the system, the PT steel frame was introduced to provide the restoring action to the system. Through the cyclic loading test, it was found that the residual displacement was only 12.9 mm. The hysteresis response of the SC-STHSW system was in a flag shape. In the study of the PT steel frame [16,17,18,20,21,31,41,51,52,53], the flag-shape hysteresis response was taken as the symbol of a system with both satisfactory self-centering capacity and sufficient energy dissipation.

With the OpenSees model was validated by the test result, four parameters, namely the initial PT stress ratio, the damper-to-wall ratio, the beam section height and the lateral resistance of the light frame wood shear wall, were considered in the parametric analysis. The last two parameters were also combined together as the lateral wall-to-frame stiffness ratio to analyze its effects. Three performance targets, including the self-centering capacity, energy dissipation and the ultimate strength, were considered in the analysis. The analysis results indicated that:

1. The increase in the initial PT stress ratio effectively increases the self-centering capacity and the ultimate strength of the SC-STHSW system. It has minor influences on the system’s energy dissipation;
2. The lower limit value of the initial PT stress ratio is suggested as 0.24 to guarantee the PT high-strength tendons’ maximum effectiveness in reducing the residual drift. However, it is not suggested to set the initial PT stress ratio larger than 0.4 in the case of the development of the plasticity in the high-strength tendons;
3. The damper-to-wall ratio has a positive effect on the increase in the energy dissipation, while it negatively affects the system’s self-centering performance. The system’s ultimate resistance is not affected by this factor;
4. The lower limit value of the damper-to-wall ratio is suggested as 0.5 if only the energy dissipation is concerned;
5. The increase in the beam section height was effective in decreasing the residual drift if the initial PT stress ratio was at a low level. It also has a positive effect on the increase in the system’s ultimate strength. No obvious relationship is found between the system’s energy dissipation and the beam section height;
6. The increase in the lateral resistance of the light frame wood shear wall shared the same function with the damper-to-wall ratio in reducing the self-centering performance of the SC-STHSW system. The use of wood shear wall with larger lateral resistance is positive in increasing the system’s energy dissipation and the ultimate strength;
7. The increase in the lateral wall-to-frame stiffness ratio increases the system’s residual drift and the energy dissipation. It has no obvious relationship with the system’s ultimate strength.

In addition, a design parameter, the self-centering ratio, was proposed for the design of the new system. The lower limit value of the self-centering ratio was suggested as 0.3 for the SC-STHSW with a lateral wall-to-frame stiffness ratio lower than 0.5. For the system with a lateral wall-to-frame stiffness ratio higher than 0.5, the lower limit value of the self-centering ratio was suggested as 0.5. In the design of the SC-STHSW system, the self-centering ratio can be used together with the lateral wall-to-frame stiffness ratio proposed by Li et al. [46].

In the future study of the SC-STHSW system, an increase in the specimens with different settings, including the different initial PT stress ratio, damper-to-wall ratio, beam section height and the usage of different light frame wood shear walls would be desirable. In this study, only three performance targets were analyzed. More performance targets and the balance between different performance targets can be explored through the parameters analysis. Furthermore, nonlinear time-history analysis of the multi-story SC-STHSW system is necessary to explore the dynamic properties of the new system.

## 5. Conclusions

In this paper, an innovative system, the SC-STHSW, is proposed. It is improved from the STHSW system by using PT technology. The experiment of one full-scale specimen was conducted to investigate the hysteretic behavior, failure modes and the PT force variation of the system. Based on the numerical model validated by the test result, a parametric analysis was conducted to explore four different parameters on the self-centering performance, energy dissipation and the ultimate strength of the system.

The experimental results showed that the residual displacement of the system was effectively controlled by using the PT steel frame. The peculiar flag shape hysteretic behavior was obtained. Moreover, there was a second increase in the system’s strength and stiffness under the large lateral deformation. After the test, the serious damages were concentrated in the damper-to-wall connections. With the application of the capacity design, the damper-to-wall connections can be designed as rigidly as possible.

The parametric analysis results revealed that the system’s self-centering performance was affected by the initial PT stress ratio *κ* and the damper-to-wall ratio *μ*. The two parameters’ influences were integrated into a design parameter, the self-centering ratio $\alpha_{E}$. It is suggested to ensure $\alpha_{E}$ is larger than 0.3 when the system’s lateral wall-to-frame stiffness ratio *λ* is lower than 0.5. When *λ* is higher than 0.5, $\alpha_{E}$ is suggested to be larger than 0.5 so that a good self-centering performance is available. As to the increase in the system’s energy dissipation, the lower limit of the damper-to-wall ratio *μ* is suggested as 0.5. The use of a wood shear wall with a large lateral resistance is also positive in increasing the system’s energy dissipation. The ultimate strength of the system is increased by the increase in the initial PT stress ratio *κ*. The increase in the beam section height, the wood shear wall’s lateral resistance and the lateral wall-to-frame ratio were also effective in increasing the system’s ultimate resistance. The presented results may serve as a technical basis for the future application of the new system.

## Figures and Tables

**Table 1:** Parameters for the characteristic strength of the damper-to-wall connection *R*_Br,k_.

*n*	*t*_1_ (mm)	*d* (mm)	*ρ*_k_ (kg/m^3^)	*f*_h,k_ (MPa)	*M*_y,Rk_ (N·mm)	*F*_ax,Rk_ (N)
38^1^	6.0	3.5	450.0^2^	53.7^3^	5990.6^4^	6188.9^5^

^1^ Note: the total screw quantity at two sides of one damper is used. ^2^ Note: the density value is taken as the common value of spruce–pine–fir (SPF) according to [40]. ^3^ Note: characteristic embedment strength is calculated based on Equation (8.15) of Eurocode 5 [38]. ^4^ Note: characteristic screw yield moment is calculated based on Equation (8.14) of Eurocode 5 [38]. ^5^ Note: characteristic withdrawal capacity is calculated based on Equation (8.38) of Eurocode 5 [38].

**Table 2:** Three light frame wood shear walls.

Wall ID	Abbreviation	Nail Spacing (mm)	*F*_Wall_ (kN)	*K*_Wall_ (kN/mm)
Wall 0	W0	75/150 (S)^1^	65	1812^2^
Wall 1	W1	75/150 (D)	82	2230
Wall 2	W2	50/100 (S)	121	3313

^1^ Note: 75/150 indicates the spacing was 75 mm along the sheathing edge and 150 mm long with the intermediate supports of the sheathings; S (or D) indicates that the wood frame is sheathed with single sided (or double sided) OSB panels with a thickness of 12 mm. ^2^ Note: the stiffness of the wood shear wall is obtained by dividing 0.4 *F*_wall_ by the corresponding displacement.

**Table 3:** Three different beam sections.

Beam ID	Abbreviation	Cross Section (mm)	*K*_Frame_ (kN/mm)
Beam 0	B0	HN400 × 150 × 8 × 13	3837^1^
Beam 1	B1	HN450 × 150 × 9 × 14	4433
Beam 2	B2	HN500 × 150 × 10 × 16	4979

^1^ Note: the stiffness of the PT frame is obtained through push over analysis. The stiffness before the gap opening was taken as *K*_Frame_.

**Table 4:** Parameters selected for the analysis.

Parameters	Symbol/Abbreviation	Level	Range	Step Size
Initial PT stress ratio	*κ*	13	0.06–0.42	0.03
Damper-to-wall ratio	*μ*	7	0.3–0.6	0.05
Light frame wood shear wall	W	3	0–2	1
Steel beam section	B	3	0–2	1
